# Rapid and Accurate Species Identification of Mitis Group Streptococci Using the MinION Nanopore Sequencer

**DOI:** 10.3389/fcimb.2020.00011

**Published:** 2020-01-30

**Authors:** Kazuo Imai, Rina Nemoto, Masahiro Kodana, Norihito Tarumoto, Jun Sakai, Toru Kawamura, Kenji Ikebuchi, Kotaro Mitsutake, Takashi Murakami, Shigefumi Maesaki, Taku Fujiwara, Satoshi Hayakawa, Tomonori Hoshino, Mitsuko Seki, Takuya Maeda

**Affiliations:** ^1^Department of Infectious Disease and Infection Control, Saitama Medical University, Saitama, Japan; ^2^Center for Clinical Infectious Diseases and Research, Saitama Medical University, Saitama, Japan; ^3^Department of Microbiology, Saitama Medical University, Saitama, Japan; ^4^Department of Laboratory Medicine, Saitama Medical University, Saitama, Japan; ^5^Department of Infectious Diseases and Infection Control, International Medical Center, Saitama Medical University, Saitama, Japan; ^6^Department of Pediatric Dentistry, Nagasaki University Graduate School of Biomedical Sciences, Nagasaki, Japan; ^7^Division of Microbiology, Department of Pathology and Microbiology, Nihon University School of Medicine, Tokyo, Japan; ^8^Division of Pediatric Dentistry, Meikai University School of Dentistry, Sakado, Japan

**Keywords:** mitis group streptococci, MinION, WIMP, Kraken, whole genome sequencing

## Abstract

Differentiation between mitis group streptococci (MGS) bacteria in routine laboratory tests has become important for obtaining accurate epidemiological information on the characteristics of MGS and understanding their clinical significance. The most reliable method of MGS species identification is multilocus sequence analysis (MLSA) with seven house-keeping genes; however, because this method is time-consuming, it is deemed unsuitable for use in most clinical laboratories. In this study, we established a scheme for identifying 12 species of MGS (*S. pneumoniae, S. pseudopneumoniae, S. mitis, S. oralis, S. peroris, S. infantis, S. australis, S. parasanguinis, S. sinensis, S. sanguinis, S. gordonii, and S. cristatus*) using the MinION nanopore sequencer (Oxford Nanopore Technologies, Oxford, UK) with the taxonomic aligner “What's in My Pot?” (WIMP; Oxford Nanopore's cloud-based analysis platform) and Kraken2 pipeline with the custom database adjusted for MGS species identification. The identities of the species in reference genomes (*n* = 514), clinical isolates (*n* = 31), and reference strains (*n* = 4) were confirmed via MLSA. The nanopore simulation reads were generated from reference genomes, and the optimal cut-off values for MGS species identification were determined. For 31 clinical isolates (*S. pneumoniae* = 8, *S. mitis* = 17 and *S. oralis* = 6) and 4 reference strains (*S. pneumoniae* = 1, *S. mitis* = 1, *S. oralis* = 1, and *S. pseudopneumoniae* = 1), a sequence library was constructed via a Rapid Barcoding Sequencing Kit for multiplex and real-time MinION sequencing. The optimal cut-off values for the identification of MGS species for analysis by WIMP and Kraken2 pipeline were determined. The workflow using Kraken2 pipeline with a custom database identified all 12 species of MGS, and WIMP identified 8 MGS bacteria except *S. infantis, S. australis, S. peroris*, and *S. sinensis*. The results obtained by MinION with WIMP and Kraken2 pipeline were consistent with the MGS species identified by MLSA analysis. The practical advantage of whole genome analysis using the MinION nanopore sequencer is that it can aid in MGS surveillance. We concluded that MinION sequencing with the taxonomic aligner enables accurate MGS species identification and could contribute to further epidemiological surveys.

## Introduction

The mitis group streptococci (MGS) comprises 17 known species: *S. pneumoniae, S. pseudopneumoniae, S. mitis, S. oralis, S. peroris, S. infantis, S. australis, S. parasanguinis, S. sinensis, S. sanguinis, S. gordonii, S. cristatus, S. massiliensis, S. rubneri, S. lactarius, S. oricebi*, and *S. panodentis*. *S. pneumoniae* is a major human pathogen associated with a wide range of infectious diseases such as pneumonia, bacteremia, meningitis, and otitis media. It is therefore important to accurately distinguish between *S. pneumoniae* and closely related species within the MGS because their pathogenic potentials and their sensitivities to various drugs differ considerably (Kitten et al., 2012; Shelburne et al., 2014). It has recently been reported that MGS has pathogenic properties that cause severe disease, especially in immunocompromised patients (Shelburne et al., 2014). Thus, it is important to accurately differentiate between MGS bacteria in routine laboratory tests to obtain accurate epidemiological information on their characteristics and to further understand the clinical significance of these species.

Identification of MGS at the species level by conventional phenotypic methods and sophisticated genetic tests targeting 16S rRNA remains unreliable (Davies et al., 2012; Jensen et al., 2016). Direct bacterial profiling via matrix-assisted laser desorption ionization-time of flight mass spectrometry (MALDI-TOF MS) has recently been introduced as a tool to enable the rapid identification of different bacteria. Unfortunately, the routine use of the Biotyper 3.0 (Bruker Daltonics GmbH, Bremen, Germany) database can result in MGS species being erroneously identified because of the similarities between their mass spectra (Ikryannikova et al., 2011; Karpanoja et al., 2014).

Multilocus sequence analysis (MLSA) via Sanger sequencing of 7 PCR products is currently the most reliable method for accurately distinguishing between MGS bacteria. MLSA requires a minimum of 7 housekeeping loci (*map, pfl, ppaC, pyk, rpoB, sodA*, and *tuf*); their relevance in taxonomic analysis has already been demonstrated (Bishop et al., 2009). Additionally, a cloud database eMLSA.net (http://viridans.emlsa.net/) has been constructed and provides a user-friendly analytical environment. However, Sanger sequencing of 7 PCR products is not suitable for routine clinical laboratories because it is expensive and time-consuming. Therefore, a suitable alternative is urgently required for clinical settings.

The MinION nanopore sequencer is a pocket-sized and USB-connected portable real-time next generation sequencer (NGS) developed by Oxford Nanopore Technologies (ONT, Oxford, UK). The main advantages of the MinION are its portability, small platform, long reads, real-time sequencing, and low-capital costs (Jain et al., 2015; Lu et al., 2016). Sequence data can be obtained in real time during generation of sequences, and MinION can be easily transported and strategically used away from laboratories in various situations. The usefulness of the MinION sequencer for the identification of bacteria species based on whole genome sequencing data has been previously reported (Tanaka et al., 2018).

What's In My Pot? (WIMP) is a cloud-based, real-time analysis and prediction system for species-level read abundance. WIMP was provided as a user-friendly pipeline based on the taxonomic classification system of Kraken (Wood and Salzberg, 2014) for MinION by ONT (Juul et al., 2015). It has been used for microbiota analysis and bacterial species identification in combination with MinION sequencing (Brown et al., 2017; Sakai et al., 2019). However, its reliability in terms of species identification, especially MGS, has not been fully discussed. In addition, WIMP cannot change the reference database to meet user requirements, unlike the local Kraken pipeline, because WIMP is provided by ONT. In this study, we evaluated the performance of whole genome sequencing by MinION for MGS species identification via WIMP and Kraken2 with the custom database adjusted for MGS species identification.

## Materials and Methods

### Bacterial Strains

In this study, 31 clinical alpha-hemolytic streptococcal strains were used. These isolates were collected from human oral mucosa from 2001 to 2004, in Tokyo, Japan. In addition, to evaluate the specificity of the test, we tested 3 different reference strains isolated by the National BioResource Project (http://www.nbrp.jp/) and the Global Bioresource Center (https://www.atcc.org/), namely *S. mitis* (JNBP 05639), *S. oralis* (JNBP 08070), and *S. pneumoniae* (ATCC 49619). *S. pseudopneumoniae* (CCUG 49455) was kindly provided by Dr. Y. Kawamura.

### MLSA Analysis

MLSA procedures based on the concatenated sequences of multiple house-keeping genes were performed according to the international scheme previously reported (Bishop et al., 2009). Briefly, PCR amplicons targeting the seven housekeeping loci were directly sequenced by the Sanger method according to the published protocol. A set of seven housekeeping genes from 12 reference strains (*S. pneumoniae, S. pseudopneumoniae, S. mitis, S. oralis, S. peroris, S. infantis, S. australis, S. parasanguinis, S. sinensis, S. sanguinis, S. gordonii*, and *S. cristatus*) for MLSA (*map, pfl, ppaC, pyk, rpoB, sodA*, and *tuf*) were obtained from the RefSeq database (*n* = 514) and were used to assign a query strain sequence for determining species clusters. The reference strains, which have been identified as MGS species based on whole-genome core sequencing-based phylogenetic analysis, were used to determine the MGS cluster (Jensen et al., 2016; Kilian and Tettelin, 2019). *S. massiliensis, S. rubneri, S. lactarius, S. oricebi*, and *S. panodentis* were excluded from this study because the reference genomes in the RefSeq database or reference MLSA phylogenetic tree were unavailable.

The accession numbers and names of the reference strains are shown in Table S1. Phylogenetic analysis and tree visualizations were conducted in MEGA7 (Kumar et al., 2016). Each concatenated sequence of the seven housekeeping genes were aligned by CLUSTAL-W. The phylogenetic tree was constructed by the Neighbor-Joining method, and the reliability of each tree topology was checked by 500 bootstrap replications.

### Nanopore Reads Simulation

The simulation of nanopore sequence reads for each reference genome obtained from the RefSeq database was generated by NanoSim-H (v 1.1.0) (Yang et al., 2017). Reads (20,000) were simulated with the R9 flow cell error model of *E. coli* with 1D chemistry (*E. coli* R9_1D) and the parameter length was 500–4,500. The reads were converted from FASTA format to FASTQ format using PyFASTAQ (v 3.17.0).

### MGS Bacteria Identification by WIMP and Kraken2

To identify the species, each dataset of simulated nanopore reads was analyzed by WIMP via EPI2ME (v 2.48) [12], and Kraken2 (Wood and Salzberg, 2014), which is a local program that assigns a taxonomy label to sequences generated by NGS. Custom databases of Kraken2 comprising 486 reference genomes of MGS obtained from the RefSeq database, which were confirmed species based on MLSA, were constructed in this study (Table S1). The taxonomy labels assigned by Kraken2 (v 2.0.8) were analyzed by Bracken (v 2.5) (Lu et al., 2017) with a parameter of 500 k-mer distribution to estimate the species-level read abundance.

To identify the MGS bacteria via WIMP and Kraken2 pipeline, the percentage of species-level read abundance to correct species taxa estimated via WIMP and Kraken2 pipeline was selected as an index. To select the optimum cut-off points of the index for the WIMP and Kraken2 pipeline, receiver-operating characteristic (ROC) analysis and Youden's index calculation were performed. The optimum cut-off value for each species was determined to maximize the Youden's index (Habibzadeh et al., 2016). All statistical analyses and the generation of heatmaps were conducted by R [v 3.4.0; R Foundation for Statistical Computing, Vienna, Austria (http://www.R-project.org/)].

### Preparation of Genomic DNA From Isolates

The Wizard Genomic DNA Purification Kit (Promega, Madison, WI) was used for DNA extraction with minor modifications. Each isolate was streaked onto sheep blood agar and incubated at 37°C with 0.5% CO_2_ overnight. Cells were harvested and suspended in 500 μL 1% NaCl, and three freeze-thaw cycles of liquid nitrogen. Mutanolysin (10,000 U/mL; 40 μL; Recenttec K. K., Tokyo, Japan) was added to the cells and the mixture was incubated for 60 min at 50°C. Thereafter, DNA was extracted via the Wizard Genomic DNA Purification Kit according to the manufacturer's instructions. Extracted DNA was purified by Agencourt AMPure XP (Beckman Coulter, Brea, CA) according to the manufacturer's instructions. The yields of extracted DNA were evaluated by NanoDrop (Thermo Fisher Scientific, Waltham, MA) and electrophoresis, and quantified with the High Sensitivity DNA kit and Qubit 2.0 Fluorometer (Thermo Fisher Scientific). Purified genomic DNA was stored at −30°C until just before use.

### MinION Sequencing

We performed whole genome sequencing of clinical isolates and reference strains using a MinION sequencer. For the multiplex and real-time MinION sequencing, a Rapid Barcoding Sequencing Kit (SQK-RBK001, ONT) was used to allow up to 12 samples to be sequenced on a single MinION Flow Cell (FLO-MIN107 R9.5 Version). Genomic DNA (each sample: 200 ng) was processed for barcoding and library preparation using the Rapid Barcoding Sequencing Kit according to the manufacturer's instructions. After running the MinION Platform QC, the DNA library was loaded into the MinION Flow Cell and the “NC_48Hr_sequencing_FLO-MIN107_SQK-RBK001_plus_basecaller” protocol was initiated using the MinKNOW ONT software (v2.0). All raw sequencing data were deposited in the Sequence Read Archive (SRA) and are available under BioProject accession number (PRJNA573516).

### Analysis of MinION Data

Local basecalling was performed using the MinKNOW ONT software (v2.0) automatically in real-time. FASTQ reads were collected 24 h after the start of sequencing. De-multiplexing and adapter trimming was performed using Porechop (v 0.2.2), and each FASTQ file obtained per ONT-barcode data was used for downstream analysis of WIMP and Kraken2 pipeline. General FASTQ read statistics were calculated by NanoPlot (v 1.13).

### Optochin Susceptibility and Bile Solubility Test

The optochin susceptibility test was performed by conventional disc diffusion protocols using optochin discs (5 μg; 6 mm; Oxoid, Hampshire, England). The discs were placed onto sheep blood agar and incubated overnight at 37°C. Isolates were considered resistant to optochin when they demonstrated inhibition zones that were smaller than 14 mm (Kellogg et al., 2001). Bile solubility tests were also performed according to the standard protocol described previously (Salo et al., 1995).

### API-20 Strep

Phenotypic identification of 12 clinical isolates by numerical profile using the API-20 Strep system (Sysmex bioMérieux, Tokyo, Japan) with Apiweb™ 1.2.1 software was carried out according to the manufacturer's instructions (French et al., 1989). Isolates were recovered and used after inoculation onto chocolate agar with sheep blood (Kyokuto Pharmaceuticals, Tokyo, Japan) at 37°C in 5% CO_2_ for 24 h.

### MALDI Biotyper Identification

MALDI-TOF MS (Bruker Daltonics GmbH) with Biotyper Real Time Classification software v3.1 (Bruker Daltonics GmbH) was also performed. Each isolate was grown on chocolate agar with sheep blood at 37°C with 5% CO_2_. From each colony, 1 μL was spotted onto MSP 48-target polished steel BC (Bruker Daltonics GmbH) to air dry at room temperature and overlaid with 1 μL of 70% formic acid before the addition of α-cyano-4-hydroxy-cinnamic acid matrix (IVD Matrix HCCA portioned) (Bruker Daltonics GmbH). The top 10 identity matches were generated with score values. Finally, the species with the highest score was artificially selected as the final identity when one or more species of bacteria were identified with scores >2.0. According to the manufacturer's recommendation, a log score value >2.0 indicated accurate identification of bacterial pathogens at the species level.

## Results

### MLSA

**Figure 1:**
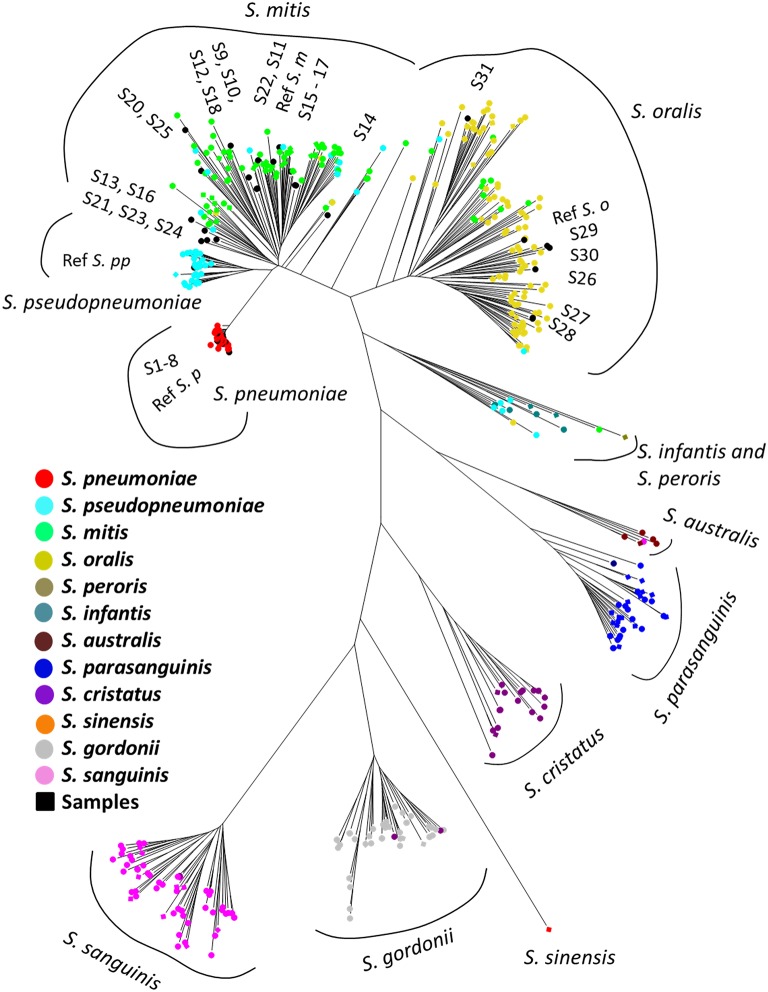
Phylogenetic analysis of mitis group streptococci based on multilocus sequence analysis. The phylogenetic tree was constructed by the Neighbor-Joining method, and the reliability of each tree topology was checked by 500 bootstrap replications. The strain names and reference numbers of reference genomes in the RefSeq database are listed in Table S1. Strains are colored according to species annotation in the RefSeq database. The detailed phylogenetic tree is shown in Figure S1.

The phylogenetic tree based on the MLSA sequence analysis of 7 house-keeping genes is shown in Figure 1 and Figure S1. A previous study showed that some reference genomes of the MGS were misidentified species deposited in the RefSeq database (Jensen et al., 2016; Kilian and Tettelin, 2019), thus we conducted species re-identification of 514 MGS reference genomes in the RefSeq database. Based on MLSA sequence analysis, the MGS could be divided into 11 clades (*S. pneumoniae* = 74; *S. pseudopneumoniae* = 40; *S. mitis* = 97; *S. oralis* = 116; *S. infantis;* and *S. peroris* = 15; *S. australis* = 5; *S. parasanguinis* = 36; *S. sinensis* = 1; *S. sanguinis* = 59; *S. gordonii* = 46; and *S. cristatus* = 25), and we found that many of the reference genomes had been mislabeled as the incorrect species at the time of registration in the RefSeq database (Table S1). The MLSA sequence analysis of 31 clinical isolates and 4 reference strains identified the species as follows: clinical isolates; *S. pneumoniae* = 8, *S. mitis* = 17, *and S. oralis* = 6, reference strains; *S. pneumoniae* = 1, *S. pseudopneumoniae* = 1, *S. mitis* = 1, and *S. oralis* = 1.

### Simulation of Nanopore Reads and Species Identification

The simulation of nanopore sequence reads set for each reference genome, which were confirmed based on MLSA in this study, were used to test for species identification based on the assigning taxonomy from each simulation read via WIMP and the Kraken2 pipeline (*S. pneumoniae* = 74; *S. pseudopneumoniae* = 40; *S. mitis* = 97; *S. oralis* = 116; *S. peroris* = 1; *S. infantis* = 14; *S. australis* = 5; *S. parasanguinis* = 36; *S. sinensis* = 1; *S. sanguinis* = 59; *S. gordonii* = 46; and *S. cristatus* = 25) (Table S1). A single type strain of *S. peroris* (ATCC 700780) assigned to the *S. infantis* and *S. peroris* cluster was treated as *S. peroris* according to the finding of a previous study via whole-genome core sequencing (Jensen et al., 2016), due to the low resolution via MLSA.

**Figure 2:**
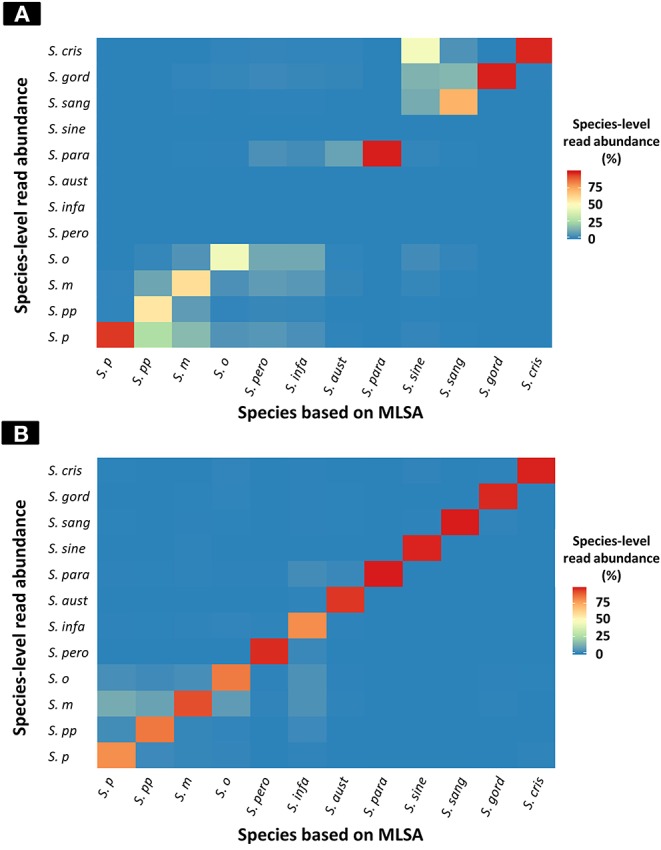
Heatmap of species-level read abundance based on WIMP and Kraken2 pipeline. Heatmaps show the species-level read abundance of simulation nanopore read sets for reference genomes in the RefSeq database. The species-level read abundance was calculated by WIMP **(A)** and Kraken2 pipeline **(B)**. Blue indicates low abundance, yellow indicates intermediate, and red indicates high. The results of the Kraken2 database showed the high species-level read abundance for species identified by multilocus sequence analysis (MLSA) among mitis group streptococci bacteria, whilst the results of WIMP showed low species-level read abundance for *S. peroris, S. infantis, S. australis*, and *S. sinensis*. *S. p, S. pneumoniae*; *S. pp, S. pseudopneumoniae*; *S. m, S. mitis*; *S. o, S. oralis*; *S. pero, S. peroris*; *S. infa, S. infantis*; *S. aust, S. australis*; *S. para, S. parasanguinis*; *S. sine, S. sinensis*; *S. sang, S. sanguinis*; *S. gord, S. gordonii*; *and S. cri, S. cristatus*.

The results of taxonomic assignment of each simulation dataset via WIMP and Kraken2 pipeline are shown in Figure 2, and Tables S2, S3. In the WIMP pipeline, *S. peroris, S. infantis, S. australis*, and *S. sinensis* were undetectable because numerous reads were erroneously assigned to *Streptococcus* sp. and *S. cristatus*, and there were no reads assigned to the correct species. However, via Kraken2 pipeline with the custom database, dominant reads among all 12 species were consistent with the results of MLSA species identification (Figure 2).

The species-level read abundance against the correct species taxon was selected as an index to determine the species of MGS. The index cut-off values for each MGS species are shown in Table 1. When we used the index to determine the MGS species, the area under the ROC curve and maximum Youden's index for each species was 1.0 (both sensitivity and specificity were 100%) for all 12 MGS species via Kraken2 pipeline, and for 8 MGS species (*S. pneumoniae, S. pseudopneumoniae, S. mitis, S. oralis, S. parasanguinis, S. sanguinis, S. gordonii*, and *S. cristatus*) via WIMP (Table 1 and Figure S2).

**Table 1:** Results of taxonomic assignment via WIMP and Kraken2 pipeline.

**Species identification based on WIMP analysis**							
		**Species-level read abundance for correct species (%)**					
**Species**	***N***	**Mean**	***SD***	**Cut-off**	**Youden's index**	**Sensitivity (%)**	**Specificity (%)**
*S. p*	74	95.60	1.68	75	1	100	100
*S. pp*	40	58.00	7.64	35	1	100	100
*S. m*	98	54.38	9.81	35	1	100	100
*S. o*	115	73.22	11.7	35	1	100	100
*S. pero*	1	Undetectable	–	Undetectable	–	–	–
*S. infa*	14	Undetectable	–	Undetectable	–	–	–
*S. aust*	5	Undetectable	–	Undetectable	–	–	–
*S. para*	36	82.02	7.93	55	1	100	100
*S. sine*	1	Undetectable	–	Undetectable	–	–	–
*S. sang*	60	77.01	3.87	55	1	100	100
*S. gord*	46	94.68	2.28	70	1	100	100
*S. cris*	24	67.55	9.60	55	1	100	100
**Species identification based on Kraken analysis**							
		**Species-level read abundance for correct species (%)**					
**Species**	***N***	**Mean**	***SD***	**Cut-off**	**Youden's index**	**Sensitivity (%)**	**Specificity (%)**
*S. p*	74	81.33	1.94	65	1	100	100
*S. pp*	40	85.22	0.58	65	1	100	100
*S. m*	98	95.99	3.46	65	1	100	100
*S. o*	115	97.00	2.95	65	1	100	100
*S. pero*	1	98.17	−	75	1	100	100
*S. infa*	14	79.93	19.18	45	1	100	100
*S. aust*	5	86.87	20.81	45	1	100	100
*S. para*	36	99.19	0.45	75	1	100	100
*S. sine*	1	98.97	−	75	1	100	100
*S. sang*	60	99.06	0.49	75	1	100	100
*S. gord*	46	98.44	0.58	75	1	100	100
*S. cris*	24	98.78	0.41	75	1	100	100

*S. p, S. pneumoniae; S. pp, S. pseudopneumoniae; S. m, S. mitis; S. o, S. oralis; S. pero, S. peroris; S. infa, S. infantis; S. aust =S. australis; S. para, S. parasanguinis; S. sine, S. sinensis; S. sang, S. sanguinis; S. gord, S. gordonii; and S. cri, S. cristatus*.

### Yield Genomic DNA and MinION Sequencing

The quality of genomic DNA yield of clinical samples and reference strains are shown in Table S4. High quality (260/280 > 1.80 and 260/230 > 2.0) and sufficient DNA (200 ng/5 μL/samples) was obtained from most of the clinical isolates and reference strains. Table 2 shows the results obtained by MinION sequencing reads from 31 isolates and 4 reference strains. Within 24 h of the run time, the median of the sequence reads allocated to each isolate by ONT-barcode was 20,513 (from 1,680 to 80,010), and the mean read length was 3,004 bp.

**Table 2:** Summary of species identification among clinical isolates.

**Isolates**	**MLSA**	**MinION**						**MALDI TOF-MS**	**Routine biochemical protocol**		
		**Total reads**	**No. of total bases**	**Mean read length**	**Coverage**	**WIMP**	**Kraken2**		**API**	**Optochin susceptibility**	**Bile solubility**
**REFERENCE STRAINS**											
ATCC 49619	*S. p*	37,731	132.4 M	3,508	60×	*S. p*	*S. p*	NA	NA	NA	NA
JNBP 05639	*S. m*	47,447	74.7 M	1,574	34×	*S. m*	*S. m*	NA	NA	NA	NA
JNBP 08070	*S. o*	49,882	93.5 M	1,875	43×	*S. o*	*S. o*	NA	NA	NA	NA
CCUG 49455	*S. pp*	7,711	25.2 M	3,267	11×	*S. pp*	*S. pp*	NA	NA	NA	NA
**CLINICAL ISOLATES**											
S1	*S. p*	18,395	31.0 M	1,684	14×	*S. p*	*S. p*	*S. p*	*S. p*	S	+
S2	*S. p*	26,806	43.9 M	1,637	20×	*S. p*	*S. p*	*S. p*	*S. p*	S	+
S3	*S. p*	27,316	127.1 M	4,654	58×	*S. p*	*S. p*	*S. p*	*S. p*	S	+
S4	*S. p*	11,765	34.9 M	2,969	16×	*S. p*	*S. p*	*S. p*	S. m*	S	+
S5	*S. p*	16,869	50.6 M	3,001	23×	*S. p*	*S. p*	*S. p*	S. m*	S	+
S6	*S. p*	36,377	167.4 M	4,602	76×	*S. p*	*S. p*	*S. p*	S. sang*	S	+
S7	*S. p*	7,579	33.2 M	4,374	15×	*S. p*	*S. p*	*S. p*	*S. p*	S	+
S8	*S. p*	22,514	70.8 M	3,143	32×	*S. p*	*S. p*	*S. p*	*S. p*	S	+
S9	*S. m*	31,807	63.5 M	1,997	30×	*S. m*	*S. m*	*S. m*	*S. m*	R	−
S10	*S. m*	20,513	79.7 M	3,884	37×	*S. m*	*S. m*	*S. m*	*S. m*	R	+^*^
S11	*S. m*	42,314	146.1 M	3,452	68×	*S. m*	*S. m*	*S. m*	S. o*	R	−
S12	*S. m*	9,642	47.8 M	4,960	22×	*S. m*	*S. m*	*S. m*	S. o*	R	+^*^
S13	*S. m*	4,259	17.2 M	4,028	8×	*S. m*	*S. m*	*S. m*	*S. m*	R	−
S14	*S. m*	10,523	31.3 M	2,977	15×	*S. m*	*S. m*	*S. m*	S. o*	R	−
S15	*S. m*	77,786	199.7 M	2,567	93×	*S. m*	*S. m*	S. p*	*S. m*	R	−
S16	*S. m*	22,768	83.8 M	3,679	39×	*S. m*	*S. m*	*S. m*	*S. m*	R	−
S17	*S. m*	4,819	15.5 M	3,223	7×	*S. m*	*S. m*	*S. m*	*S. m*	R	−
S18	*S. m*	80,010	208.0 M	2,599	97×	*S. m*	*S. m*	*S. m*	*S. m*	R	−
S19	*S. m*	52,061	200.3 M	3,848	93×	*S. m*	*S. m*	*S. m*	*S. m*	R	−
S20	*S. m*	14,388	64.1 M	4,453	30×	*S. m*	*S. m*	*S. m*	*S. m*	R	−
S21	*S. m*	13,395	51.4 M	3,836	24×	*S. m*	*S. m*	*S. m*	S. o*	R	−
S22	*S. m*	6,614	14.2 M	2,141	7×	*S. m*	*S. m*	S. o*	G. m*	R	−
S23	*S. m*	14,644	32.7 M	2,233	15×	*S. m*	*S. m*	*S. m*	*S. m*	R	−
S24	*S. m*	11,277	266.5 M	4,993	124×	*S. m*	*S. m*	*S. m*	*S. m*	R	−
S25	*S. m*	23,957	37.8 M	1,579	18×	*S. m*	*S. m*	*S. m*	*S. m*	R	−
S26	*S. o*	45,499	128.7 M	2,829	64×	*S. o*	*S. o*	*S. o*	*S. o*	R	−
S27	*S. o*	10,793	22.7 M	2,101	11×	*S. o*	*S. o*	*S. o*	*S. o*	R	−
S28	*S. o*	15,630	48.0 M	3,068	24×	*S. o*	*S. o*	*S. o*	*S. o*	R	−
S29	*S. o*	1,680	2.5 M	1,461	1×	*S. o*	*S. o*	*S. o*	S. m*	R	−
S30	*S. o*	6,548	5.2 M	786	3×	*S. o*	*S. o*	*S. o*	S. m*	R	−
S31	*S. o*	25,997	56.5 M	2,174	28×	*S. o*	*S. o*	*S. o*	*S. o*	R	−

S. p, S. pneumoniae; S. pp, S. pseudopneumoniae; S. m, S. mitis; S. o, S. oralis; S. sang, S. sanguinis; and G. m, G. morbillorum.

*Asterisk indicates discrepancy in the results of multilocus sequence analysis as a reference identification method compared with other methods*.

### Species Identification of Clinical Samples via WIMP and Kraken2

**Figure 3:**
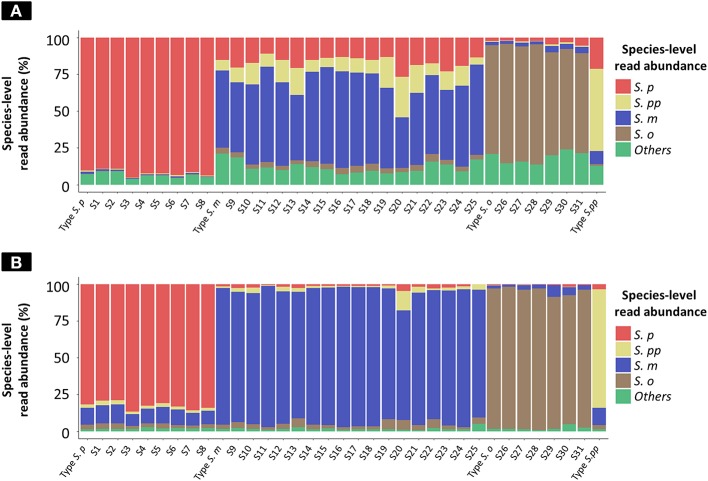
Species-level read abundance based on WIMP and Kraken2 pipeline for clinical isolates and reference strains. The stacked bar graphs show the species-level read abundance in MinION sequencing reads (Kilian and Tettelin, 2019); abundance was calculated based on WIMP **(A)** and Kraken2 pipeline **(B)**. *S. p, S. pneumoniae*; *S. pp, S. pseudopneumoniae*; *S. m, S. mitis*; *S. o, S. oralis*; others*, S. peroris, S. infantis, S. australis, S. parasanguinis, S. sinensis, S. sanguinis, S. gordonii, S. cristatus*, and *Streptococcus*. sp.

The results of taxonomy assignment of each clinical isolate and reference strain (*S. pneumoniae* = 9, *S. mitis* = 18, *S. oralis* = 7, and *S. pseudopneumoniae* = 1) via WIMP and Kraken2 pipeline are shown in Figure 3 and Supplementary Tables S2, S3. The average frequencies of taxonomy assignment against correct reference species among clinical isolates and reference strains were as follows: *S. pneumoniae* = 92.18% (SD = 2.05%), *S. pseudopneumoniae* = 56.05%, *S. mitis* = 55.40% (SD = 8.33%), and *S. oralis* = 74.58% (SD = 6.00%) in WIMP workflow, and *S. pneumoniae* = 82.68% (SD = 2.68%), *S. pseudopneumoniae* = 81.69%, *S. mitis* = 90.38% (SD = 5.00%), and *S. oralis* = 93.29% (SD = 3.37%) in Kraken2 pipeline. The MGS species identified among clinical isolates and reference strains by MinION sequencing via WIMP and Kraken2 pipeline with the cut-off value determined in this study were consistent with those identified in MLSA analysis.

### Comparison of Species Identification Methods

Table 2 shows the results of the species identification method by MALDI Biotyper, biochemical method, MLSA, and MinION-based methods for 31 clinical isolates. Using the API-20 Strep system, 5 isolates were confirmed as *S. pneumoniae*, 16 as *S. mitis*, 8 as *S. oralis*, 1 as *S. sanguinis*, and 1 as *Gemella morbillorum* among the 31 clinical isolates. The detailed biochemical and enzymatic profiles of these strains are shown in Table S5. There were some discrepancies between MLSA as a reference method and the API-20 Strep system among 3 of the *S. pneumoniae* strains, which were misidentified in Api-20 Strep tests as *S. mitis* (S4 and 5) and *S. sanguinis* (S6), among 4 of the *S. mitis* strains, which were misidentified in API-20 Strep tests as *S. oralis* (S11-12 and 14) and *G. morbillorum* (S22), and among 2 *S. oralis* strains, which were misidentified in Api-20 Strep tests as *S. mitis* (S29 and 30). In this study, all *S. pneumoniae* isolates were sensitive to optochin and positive in the bile solubility test, whereas 2 isolates (S10 and 12) were also positive in the bile solubility test.

All *S. pneumoniae* and *S. oralis* isolates were correctly identified by the MALDI Biotyper as the top choice among strains with log score values of 2.0 or more (scores 2.106–2.25). A total of 15 isolates of 17 *S. mitis* were also correctly identified at the species level (Table 2), whereas 2 strains of *S. mitis* (S15 and 22) were misidentified as *S. pneumoniae* and *S. oralis*, respectively. However, the top 10 results in 14 of 15 isolates of *S. mitis* that were correctly identified by MALDI Biotyper contained at least one significant mismatch with a log score value of 2.0 or more. All data are presented in Table S6.

## Discussion

Currently, routine biochemical protocols for species identification are not accurate enough to allow differentiation between MGS species (Davies et al., 2012). Our results proved the limitation of the API-20 Strep system, optochin susceptibility test, and bile solubility test for MGS species identification, as shown in the previous study (Davies et al., 2012). While the available databases for MALDI Biotyper have improved in recent years, the technology still results in misidentifications among MGS species. In fact, our study results showed inaccurate MGS species identification via this protocol. Despite the benefits of MALDI Biotyper in the identification of various bacteria, such as rapid processing, reasonable running costs, and simple operating procedures, the initial high cost of implementing it is an obstacle to its popularity in various clinical environments (Harju et al., 2017).

Identification of *S. pneumoniae* to distinguish it from closely related MGS is not always accurate in clinical settings when using conventional bacterial tests such as the optochin susceptibility test and the bile solubility test (Pinto et al., 2013). In recent years, conventional PCR or real-time PCR assay have been widely used for autolysin (*lytA*), pneumolysin (*ply*), pneumococcal surface antigen A (*psaA*), and a DNA fragment of unknown function (*Spn9802*) (Jado et al., 2001; Llull et al., 2006; Stralin et al., 2014). Moreover, the World Health Organization recommends the real-time PCR assay for *lytA* (Satzke et al., 2013). However, qualitative conventional PCR methods do not completely distinguish between *S. pneumoniae* and MGS species (Simoes et al., 2016).

Phylogenetic analyses based on MLSA and the whole-genome core sequencing including core-genome MLST (Maiden et al., 2013) via NGS data are the most reliable methods for MGS species identification (Jensen et al., 2016). In particular, whole-genome core sequencing enables more high-resolution phylogenetic analyses of MGS bacteria than MLSA (Jensen et al., 2016). However, whole-genome core sequencing requires sequence data with a low error rate and enough coverage to assemble the accurate whole genomes of the bacteria. An NGS such as the Illumina platform (Illumina, San Diego, CA) is suitable for the whole-genome core sequencing due to its high accuracy. However, it has high initial costs, requires trained personnel, and is a time-consuming process. Therefore, it is a challenge to establish a facility with an Illumina platform for clinical settings. MinION is a portable and real-time NGS, but sequencing reads have a high error rate with 1D chemistry (~ 11%) and 1D^2^ chemistry (~ 7%) (Imai et al., 2018), and the assembled bacteria genome reads contain many mismatches and insertions and deletions (Lu et al., 2016; Goldstein et al., 2019). Therefore, MinION sequencing is thought to be unsuitable for phylogenetic analyses based on core-genome MLST. Further study is needed to evaluate the accuracy of core-genome MLST using assembly data composed only by MinION sequencing.

Average nucleotide identity (ANI) is an alternative method to DNA–DNA hybridization based on NGS sequencing data for the identification of bacteria species (Konstantinidis and Tiedje, 2005; Goris et al., 2007). Tanaka et al. showed that MinION sequencing is applicable for ANI analysis of *Vibrionaceae*, and sequencing reads with high error rates do not affect the ANI result (Tanaka et al., 2018). However, ANI has been previously shown to be unsuitable for distinguishing between MGS species because specific levels of identity were not defined (Jensen et al., 2016).

In this study, we showed that a species identification method based on the taxonomic classification system of Kraken2 pipeline can distinguish between MGS species with high sensitivity and specificity. This method directly uses long sequencing reads generated by MinION, which has the ability to distinguish between MGS species. Our results showed that sequencing reads with high error rates do not affect the results of species identification via WIMP and Kraken2 pipeline based on the experiment using simulation nanopore reads with 1D chemistry generated from reference genomes of the MGS and raw nanopore reads generated by clinical isolates and reference strains.

WIMP uses Kraken pipeline and the database comprises reference sequences in the RefSeq database (Juul et al., 2015). WIMP database is not open to the public and seems to be composed of reference genomes that are defined as assembly level “complete” in the RefSeq database. Currently, the complete genomes of only 7 MGS species (*S. pneumoniae, S. pseudopneumoniae, S. mitis, S. oralis, S. gordonii, S. sanguinis*, and *S. parasanguinis*) are available in the RefSeq database. Therefore, WIMP can detect more than seven species but it cannot detect other MGS species (*S. sinensis, S. peroris, S. infantis*, and *S. australis*). In addition, we showed that some reference genomes of MGS were deposited as incorrect species in the RefSeq database, as mentioned in the previous study (Jensen et al., 2016; Kilian and Tettelin, 2019). Regarding *S. mitis* and *S. pseudopneumoniae*, the accuracy of taxonomic assignments was lower than that for other MGS species when using only WIMP. Therefore, the WIMP database may be composed of reference genomes that are deposited as incorrect species. To solve these problems, we assigned reference genomes of the MGS as correct species by MLSA, and then made a custom database via Kraken2, which was composed of all reference genomes of the MGS including *S. sinensis, S. peroris, S. infantis*, and *S. australis*. The Kraken2 pipeline with custom database enabled the identification of all MGS species and contributed to the improved and accurate taxonomic assignment compared with WIMP, especially for *S. mitis* and *S. pseudopneumoniae*. Modification of the RefSeq database is needed for further study including proper MGS identification.

Gram-positive cocci of MGS are recalcitrant during lysis in the purification of long-read DNA (Yuan et al., 2012). Low molecular weight, incorrectly quantified, and/or contaminated DNA and various substances (e.g., proteins, organic solvents) injected into the MinION flow cell directory could have a significant impact on the sequence runs and total reads obtained. The protocol, including mutanolysin treatments, has a significant effect on DNA quality and procedures via the Rapid Barcoding Sequencing Kit. Furthermore, the protocol for the DNA extraction and library preparation for suitable MinION sequencing was performed within 3.5 h; real-time analysis could further shorten the time and would take significantly less time than MLSA analysis. In this study, the results of the identification of the MGS species using the WIMP and Kraken2 pipelines showed that the MGS species were correctly identified even in samples with low coverage (>1×), whereas whole-genome core sequencing and ANI, which use assembly data, generally need high coverage of nanopore reads (coverage >20×) (Lu et al., 2016). Presently, the Flongle Flow Cell sequencing device, which is a low-priced device with 126 channels, compared with 512 channels in the MinION, is available and will provide further versatility and reduced running costs.

A limitation of this study is the small number of clinical isolates, so these isolates are not completely representative of clinical isolates in Japan or other countries. A large number of clinical isolates, a variety of species, and genetic diversity are still needed to evaluate the pipeline and DNA extraction method from MGS. The single type strain of *S. peroris* was available in the RefSeq database and was not discernable from *S. infantis* by MLSA and whole-genome core sequencing analysis because of sequence similarities; however, *S. peroris* showed a different biochemical character and DNA–DNA hybridization score from *S. infantis* (Kawamura et al., 1998; Jensen et al., 2016). The addition of more reference strains of *S. peroris* and further study are needed to verify the accuracy of species identification of *S. peroris* and *S. infantis* using our methods. In addition, MinION sequencing requires complex DNA extraction modules, library preparation kits, and flow cells, making this method more expensive than MALDI-TOF MS and traditional biochemical methods. Further improvements to our method are still needed to reduce costs.

Thus, we conclude that whole genome analysis using MinION sequencing with taxonomic classification software of Kraken2 pipeline with a custom database adjusted for MGS species identification allows accurate identification of 12 MGS species. WIMP has limited ability to identify MGS isolates, although it can identify *S. pneumoniae, S. pseudopneumoniae, S. mitis*, and *S. oralis*. Modification of the WIMP database is required for the accurate identification of MGS species. Our method using Kraken2 pipeline could contribute to obtaining accurate epidemiological information on the characteristics of MGS bacteria in clinical settings.

## Data Availability Statement

The datasets generated for this study can be found in NCBI, accession number PRJNA573516.

## Author Contributions

MS and TMa designed the experiments. KIm, RN, MK, NT, JS, TK, and TMa performed the experiments. KIk, KM, TF, TH, TMu, SH, and SM provided scientific guidance. KIm, KM, NT, TMu, SM, MS, and TMa prepared the manuscript.

### Conflict of Interest

The authors declare that the research was conducted in the absence of any commercial or financial relationships that could be construed as a potential conflict of interest.

## Supplementary Material

Figure S1 Detailed phylogenetic analysis of mitis group streptococci based on MLSA. The strain name and reference number of reference genomes in the RefSeq database are shown in the phylogenetic tree. Species were annotated in the RefSeq database. The phylogenetic tree was constructed by the Neighbor-Joining method, and the reliability of each tree topology was checked by 500 bootstrap replications. Squares indicate the reference strains previously identified as mitis group streptococci species based on the whole-genome core sequencing-based phylogenetic analysis (Jensen et al., 2016; Kilian and Tettelin, 2019).

Figure S2 ROC curve of WIMP and Kraken2. ROC curve of WIMP **(A)** and Kraken2 pipeline **(B)** for species identification of mitis group streptococci bacteria, conducted in simulated nanopore read sets generated from reference genomes. *S. p, S. pneumoniae*; *S. pp, S. pseudopneumoniae*; *S. m, S. mitis*; *S. o, S. oralis*; *S. pero, S. peroris*; *S. infa, S. infantis*; *S. aust, S. australis*; *S. para, S. parasanguinis*; *S. sine, S. sinensis*; *S. sang, S. sanguinis*; *S. gord, S. gordonii*; *and S. cri, S. cristatus*.

Table S1 List of reference genomes in the RefSeq database.

Table S2 Summary of species identification via WIMP.

Table S3 Summary of species identification via Kraken2 pipeline.

Table S4 Quality of genomic DNA extracted from clinical isolates and reference strains.

Table S5 Detailed biochemical and enzymatic profiles of clinical isolates in API 20 Strep.

Table S6 Top 10 score values of bacterial species in MALDI-TOF MS with Biotyper Real Time Classification software v3.1.
