# Supplementary figures and images for: Rapid and Accurate Species Identification of Mitis Group Streptococci Using the MinION Nanopore Sequencer
Source: Front Cell Infect Microbiol. 2020 Jan 30;10:11. doi: 10.3389/fcimb.2020.00011 (PMC7002467; doi:10.3389/fcimb.2020.00011)

Fig. S1

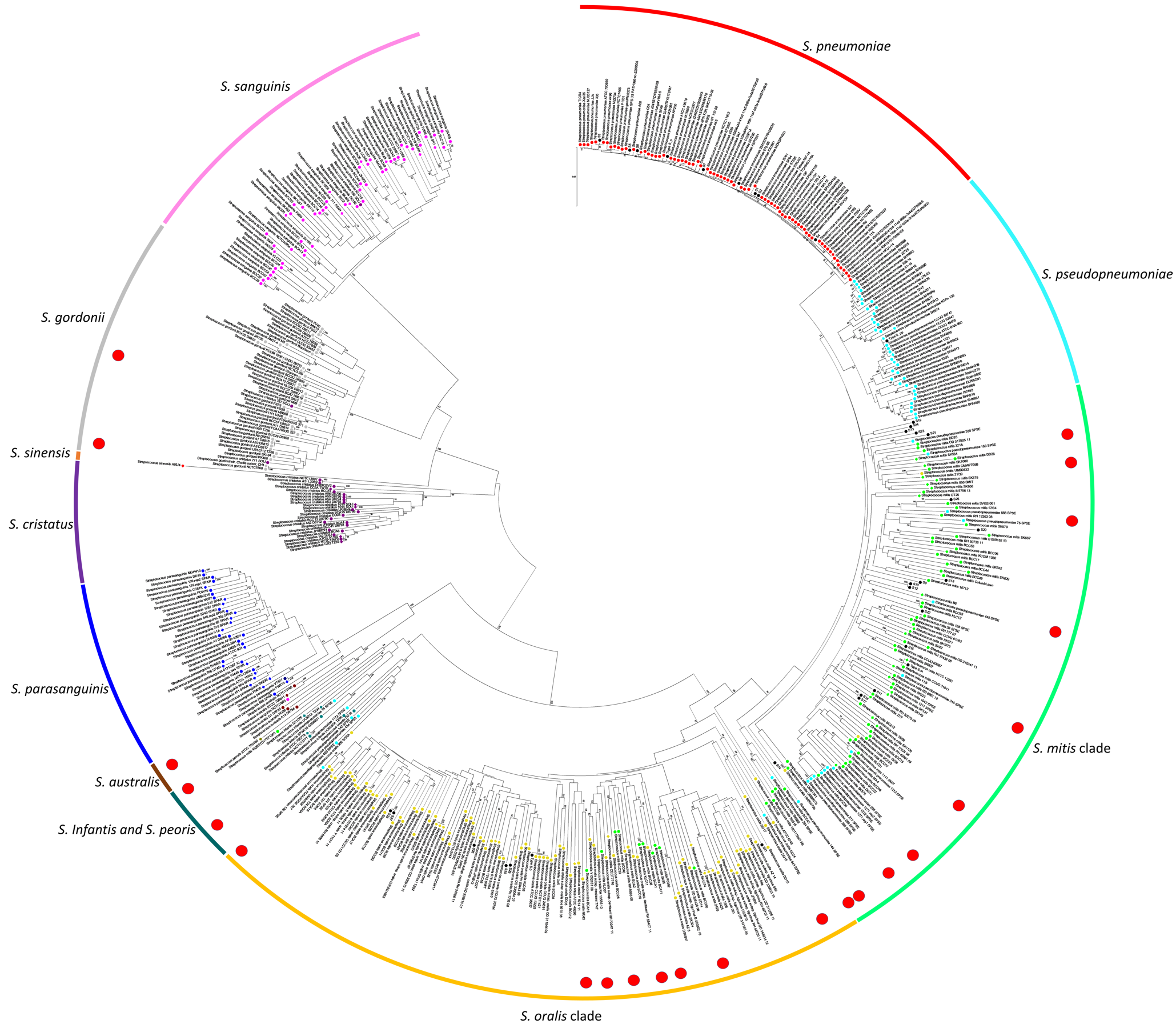

Supplement: Figure S1 — Detailed phylogenetic analysis of mitis group streptococci based on MLSA. The strain name and reference number of reference genomes in the RefSeq database are shown in the phylogenetic tree. Species were annotated in the RefSeq database. The phylogenetic tree was constructed by the Neighbor-Joining method, and the reliability of each tree topology was checked by 500 bootstrap replications. Squares indicate the reference strains previously identified as mitis group streptococci species based on the whole-genome core sequencing-based phylogenetic analysis (Jensen et al., 2016; Kilian and Tettelin, 2019). [file Data_Sheet_1.PDF]

**A**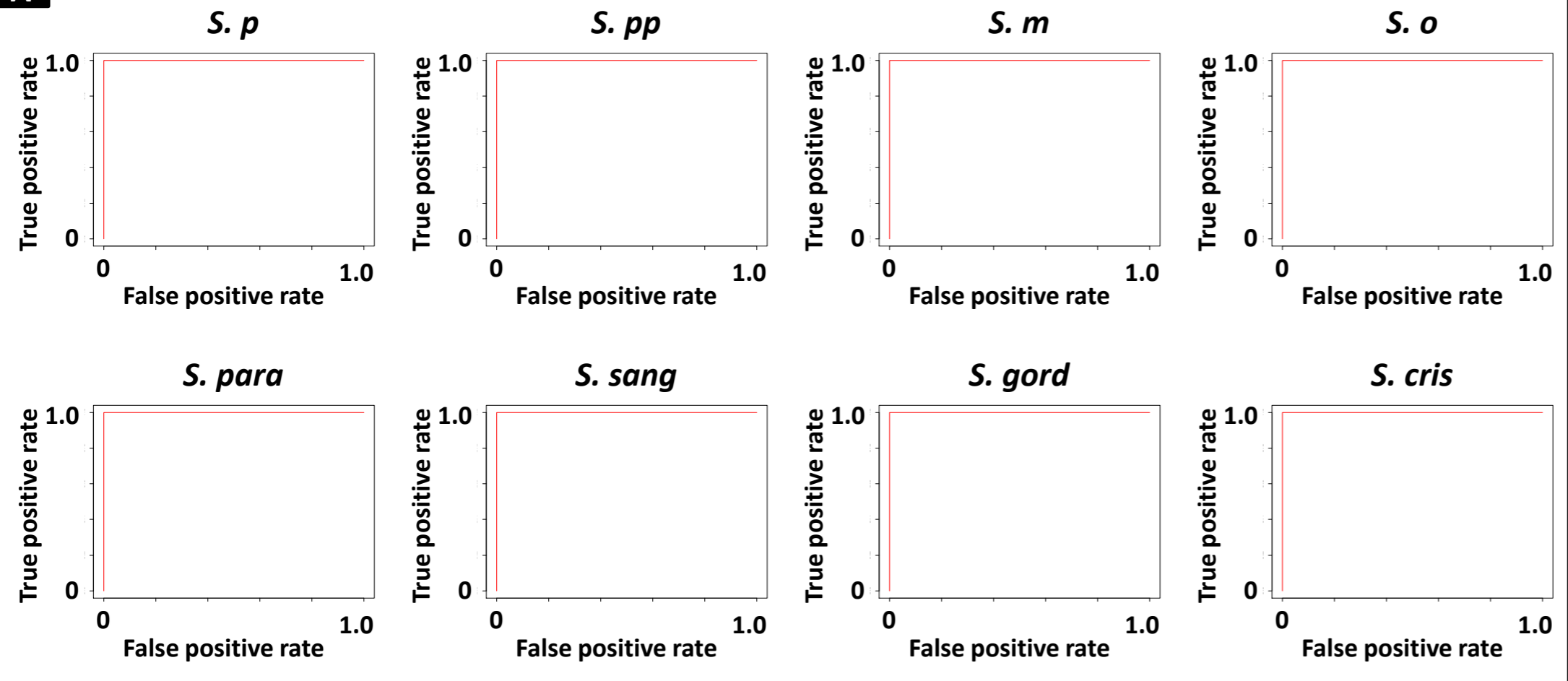**B**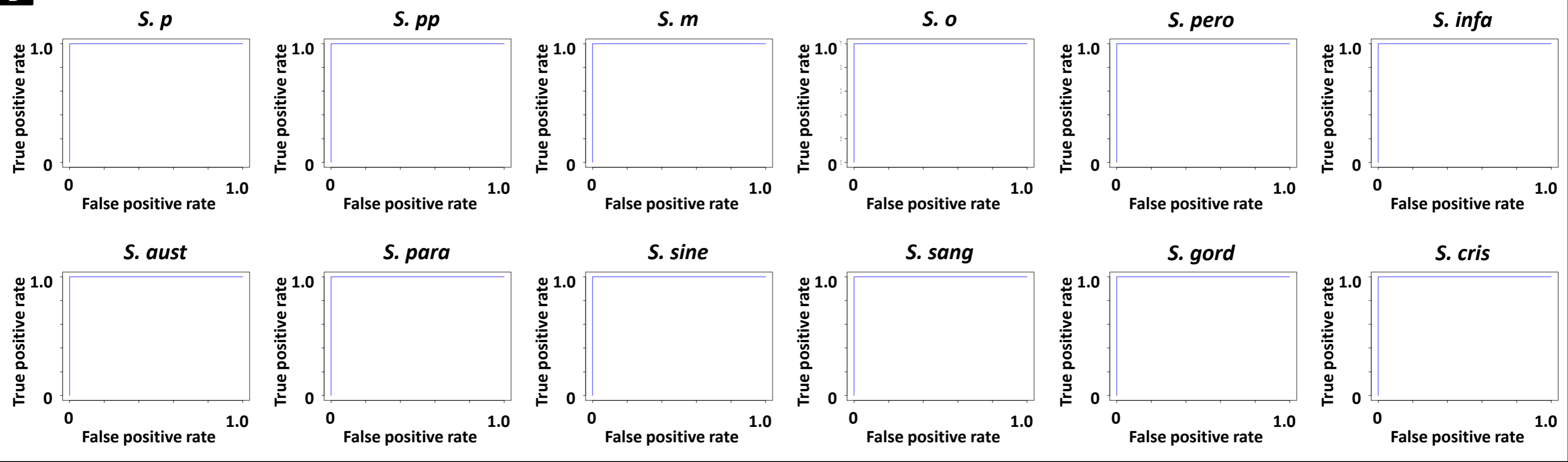

Supplement: Figure S2 — ROC curve of WIMP and Kraken2. ROC curve of WIMP (A) and Kraken2 pipeline (B) for species identification of mitis group streptococci bacteria, conducted in simulated nanopore read sets generated from reference genomes. S. p, S. pneumoniae; S. pp, S. pseudopneumoniae; S. m, S. mitis; S. o, S. oralis; S. pero, S. peroris; S. infa, S. infantis; S. aust, S. australis; S. para, S. parasanguinis; S. sine, S. sinensis; S. sang, S. sanguinis; S. gord, S. gordonii; and S. cri, S. cristatus. [file Data_Sheet_2.PDF]
